# O-GlcNAcylation and Inflammation: A Vast Territory to Explore

**DOI:** 10.3389/fendo.2014.00235

**Published:** 2015-01-09

**Authors:** Léa Baudoin, Tarik Issad

**Affiliations:** ^1^UMR8104, CNRS, Institut Cochin, Université Paris Descartes, Paris, France; ^2^U1016, INSERM, Paris, France

**Keywords:** *O*-GlcNAc glycosylation, diabetes, metabolic syndrome, inflammation, cytokines, macrophages, nitric oxide, NFκB

## Abstract

O-GlcNAcylation is a reversible post-translational modification that regulates the activities of cytosolic and nuclear proteins according to glucose availability. This modification appears to participate in several hyperglycemia-associated complications. An important feature of metabolic diseases such as diabetes and obesity is the presence of a low-grade chronic inflammation that causes numerous complications. Hyperglycemia associated with the metabolic syndrome is known to promote inflammatory processes through different mechanisms including oxidative stress and abnormally elevated protein O-GlcNAcylation. However, the role of O-GlcNAcylation on inflammation remains contradictory. O-GlcNAcylation associated with hyperglycemia has been shown to increase nuclear factor κB (NFκB) transcriptional activity through different mechanisms. This could contribute in inflammation-associated diabetic complications. However, in other conditions such as acute vascular injury, O-linked *N*-acetyl glucosamine (*O*-GlcNAc) also exerts anti-inflammatory effects via inhibition of the NFκB pathway, suggesting a complex regulation of inflammation by *O*-GlcNAc. Moreover, whereas macrophages and monocytes exposed to high glucose for a long-term period developed a pro-inflammatory phenotype, the impact of O-GlcNAcylation in these cells remains unclear. A future challenge will be to clearly establish the role of O-GlcNAcylation in pro- and anti-inflammatory functions in macrophages.

## Introduction

In the last decades, changes in lifestyle, including excessive energy intake and consumption of food enriched in saturated fat, combined with the lack of physical activity, have led to a dramatic increased prevalence of pathologies such as diabetes, obesity, and atherosclerosis. These pathologies are part of the metabolic syndrome, which constitutes one of the major threats to global health.

It is now well established that these metabolic diseases are associated with a low-grade chronic inflammation (1) that causes complications such as nephropathy, neuropathy, retinopathy, and atherosclerosis, and contributes to morbidity and mortality associated with the metabolic syndrome. This low-grade inflammation is characterized by an abnormal cytokine production. Thus, it has been demonstrated that the adipose tissue of obese individuals produce higher levels of the pro-inflammatory cytokine tumor-necrosis factor α (TNFα) and other pro-inflammatory factors such as interleukin (IL) 6 (1). The excessive amount of nutritional lipids might have a role not only in the pathogenesis of obesity-associated insulin resistance but also in the chronic inflammation associated with this condition. Indeed, free fatty acids can activate the lipopolysaccharide (LPS) receptor toll-like receptor (TLR) 4 and induce the production of pro-inflammatory cytokines by macrophages (2). Not only lipids but also high-glucose concentrations are involved in inflammatory processes (3, 4). High glycemic index diets appeared to play a key role in the establishment and persistence of inflammation (5–7). In contrast, a 4 weeks food restriction in obese patients was sufficient to significantly reduce oxidative stress (8).

**Figure 1:**
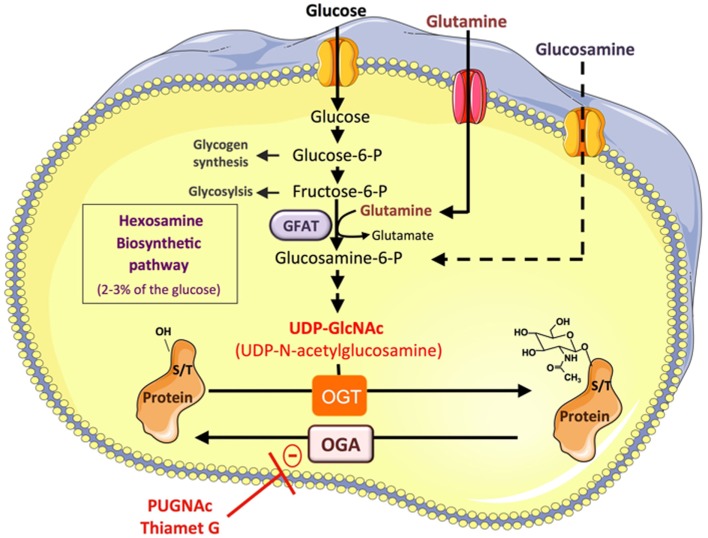
**Protein O-GlcNAcylation depends on the flux of glucose through the hexosamine biosynthesis pathway**. A small fraction of the glucose entering the cell feeds the hexosamine biosynthetic pathway (HBP) to produce UDP-GlcNAc, the substrate used by *O*-GlcNAc-transferase (OGT) to add *N*-acetyl glucosamine on serine or threonine residues of cytosolic or nuclear proteins. This dynamic and reversible post-translational modification controls the activity, the localization, or the stability of proteins according to glucose availability. Glucose enters the HBP as fructose-6-phosphate. The latter is converted to glucosamine-6-phosphate by the glutamine:fructose-6-phosphate amidotransferase (GFAT), the rate limiting enzyme of the pathway. After a subset of reactions, UDP-*N*-acetylglucosamine (UDP-GlcNAc) is generated and used by OGT to add GlcNAc on serine or threonine residues of target proteins. The *O*-GlcNAc moiety is removed from *O*-GlcNAc-modified proteins by the *O*-GlcNAcase (OGA). Experimentally, O-GlcNAcylation of proteins can be increased by incubating the cells with high concentrations of glucose, or with glucosamine, which bypass the rate limiting step catalyzed by GFAT. Inhibitors of OGA such as O-[2-acetamido-2-deoxy-d-glucopyranosylidene] amino-*N*-phenylcarbamate (PUGNAc) or (3aR,5R,6S,7R,7aR)-2-(ethylamino)-3a,6,7,7a-tetrahydro-5-(hydroxymethyl)-5H-Pyrano[3,2-d]thiazole-6,7-diol (Thiamet-G) can also be used to increase the *O*-GlcNAc level on proteins.

It is well documented that hyperglycemia associated with the metabolic syndrome promotes abnormally elevated protein O-GlcNAcylation, which participates in the glucotoxicity phenomenon (9). O-GlcNAcylation is a reversible post-translational modification consisting in the addition of *N*-acetylglucosamine to serine or threonine on cytosolic and nuclear proteins (Figure 1). Only two enzymes, *O*-GlcNAc transferase (OGT) and *O*-GlcNAcase (OGA), control the level of O-linked *N*-acetyl glucosamine (*O*-GlcNAc) on proteins. OGT uses UDP-GlcNAc, produced through the hexosamine biosynthetic pathway (HBP) to *O*-GlcNAcylate proteins, whereas OGA removes *O*-GlcNAc from proteins. Thus, according to glucose availability and its flux through the HBP, O-GlcNAcylation modulates protein functions by regulating their sub-cellular localization, stability, interaction with protein partners, or activity. More than 1000 proteins have now been identified as target of this modification, including transcription factors (10–17) and signaling molecules (9, 18–22) involved in glucose and lipid metabolism, insulin resistance, and inflammation. In addition to glucose, the *O*-GlcNAc also includes amine and acetyl moieties, and therefore also integrates amino-acids (glutamine) and fatty acid (AcetylCoA) metabolisms, suggesting that the availability of other nutrients may also be sensed by this pathway. Thus, infusion of a lipid emulsion in rats induced a twofold increase in UDP-GlcNAc content in skeletal muscle, associated with insulin resistance. Moreover, fatty acids can directly regulate the expression of glutamine:fructose-6-phosphate amidotransferase (GFAT) (23) and other enzymes of the HBP pathway (24) in muscle and pancreatic β-cell. Therefore, increased nutrients, and particularly increased blood glucose and fatty acids levels associated with excess food intake, obesity, and/or diabetes, are likely to impact numerous cellular processes, including those involved in inflammation, through protein O-GlcNAcylation.

## O-GlcNAcylation, Diabetic Complications, and Inflammatory Processes

A number of experimental data have suggested the involvement of the HBP in pathological manifestations of the metabolic syndrome, such as diabetic associated-kidney disease. Indeed, one-third of diabetic patients will develop diabetic nephropathy, a chronic microvascular complication leading to a progressive decline in renal function, decreased glomerular filtration rate and proteinuria. Clinical trials have demonstrated that high glucose is central to the pathogenesis of diabetic nephropathy (25), and the beneficial effect of glycemia correction on renal complications has been demonstrated (26). Mesangial cells are smooth muscle-like pericytes that surround the filtration capillaries within glomerulus (27). In these cells, glucose flux, through the HBP pathway, regulates the expression of pro-fibrotic factors such as transforming growth factor β1 (TGFβ1) and plasminogen activator inhibitor 1 (PAI-1), and extracellular matrix components (28, 29), at least in part via the O-GlcNAcylation of transcription factors such as Sp1 (11, 30). In mesangial cells, the HBP pathway also regulates the expression of pro-inflammatory factors such as vascular cell adhesion molecule-1 (VCAM-1), IL6, and TNFα, through the nuclear factor κB (NFκB) pathway (31). Abnormal activation of the NFκB pathway is certainly a major contributor in inflammation-associated diabetic complications. In vascular smooth muscle cells, high-glucose conditions resulted in NFκB activation (32). Peripheral blood mononuclear cells isolated from patients with diabetic nephropathy showed an increased activation of NFκB that could be corrected by anti-oxidant treatment (33, 34). Glucose oxidative stress is obviously central to glucotoxicity in diabetic conditions (35), and a link between hyperglycemia, oxidative stress, and O-GlcNAcylation has been proposed, reinforcing the potential involvement of O-GlcNAcylation in inflammation (36, 37). Therefore, exploring the potential regulation of NFκB activity by O-GlcNAcylation in different settings is of paramount importance.

## O-GlcNAcylation and the NFκB Pathway

**Figure 2:**
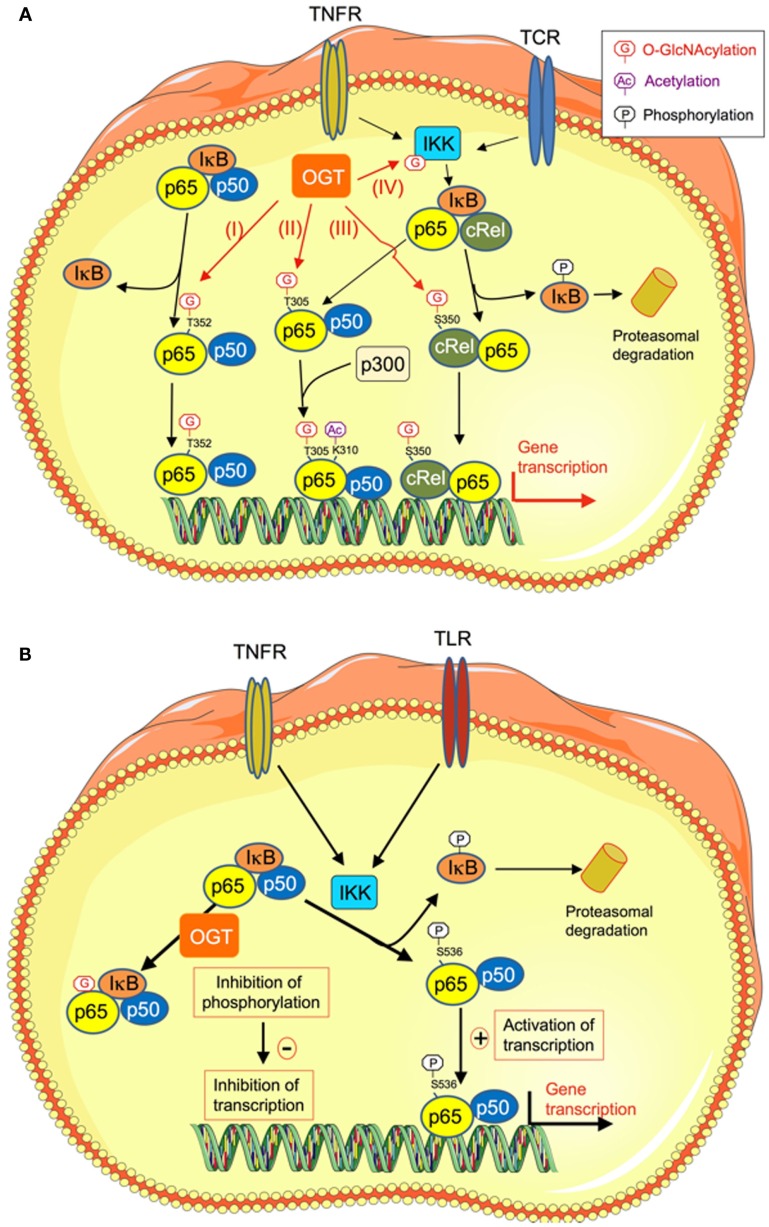
**O-GlcNAcylation regulates NFκB transcriptional activity through different mechanisms**. **(A)** O-GlcNAcylation stimulates NFκB transcriptional activity. High-glucose conditions are known to promote inflammatory processes through different mechanisms, including increased O-GlcNAcylation of NFκB. Several mechanisms have been described that could account for increased transcriptional activity of this factor upon O-GlcNAcylation. (I) O-GlcNAcylation of p65/RelA on T352 decreases its affinity for IκB, resulting in increased in its nuclear localization and transcription of its target genes (41). (II) O-GlcNAcylation of T305 on RelA promotes NFκB transcriptional activity by potentiating its p300-dependent acetylation on K310 (42). (III) O-GlcNAcylation of c-Rel on S350. This modification increases c-Rel DNA binding and transcriptional activity. (IV) O-GlcNAcylation of the β-subunit of IKK on Ser733 stimulates its activity, resulting in increased phosphorylation and degradation of IκB, and thereby increased NFκB activity. **(B)** O-GlcNAcylation inhibits NFκB transcriptional activity. O-GlcNAcylation-inducing treatments appear to have anti-inflammatory and vaso-protective effects during acute vascular injury. In rat aortic smooth muscle cells, O-GlcNAcylation of NFκB specifically inhibits its phosphorylation on Ser 536, while leaving other phosphorylation sites unaffected. This results in increased NFκB binding to IkB, inhibition of TNFα-induced NFκB DNA binding, and reduction of expression of genes coding for inflammatory mediators (TNFR, TNFα receptor; TCR, T-cell receptor; IKK, Iκ kinase).

The transcription factor NFκB is involved in a large number of cell functions including apoptosis, cell survival, and differentiation, and is critical to immune response and inflammation. NFκB family comprises five proteins, p65 (RelA), RelB, c-Rel, p105/p50 (NFκB1), and p100/52 (NFκB2) that associate to form distinct homo and hetero-dimeric complexes (38–40). In non-stimulated cells, NFκB is inactive and is retained in the cytoplasm by the inhibitor of κB (IκB) (Figure 2). Upon stimulation by pro-inflammatory cytokines, LPS, or growth factors, IκB is phosphorylated by the IκB kinase (IKK). This phosphorylation leads to IκB ubiquitination and proteosomal degradation. Free NFκB can then translocate into the nucleus to activate its target genes (38–40).

Nuclear factor κB activation has been implicated in the metabolic syndrome and in diabetes pathogenesis (43–46). Because NFκB is mainly regulated by post-transcriptional modifications (with an important role of phosphorylation and acetylation), and because high glucose is known to activate NFκB and stimulate its target genes, different studies focused on the potential role of *O*-GlcNAc on NFκB activation.

### O-GlcNAcylation as a positive regulator of NFκB activity

In the first study addressing this question, mesanglial cells treated with glucosamine or high-glucose exhibited an increased nuclear protein binding to NFκB consensus sequences in an electromobilty shift assay, correlated with O-GlcNAcylation of p65 (31). This observation suggested that NFκB O-GlcNAcylation could play a part in inflammatory processes. However, in that first study, the *O*-GlcNAc modification sites on NFκB had not been identified and the mechanism by which *O*-GlcNAc modification led to NFκB activation remained unclear (31).

It now clearly appears that different mechanisms, acting at various cellular levels, are involved in the effects of O-GlcNAcylation on activation of NFκB signaling. First, O-GlcNAcylation can regulate the interaction between NFκB and its inhibitor IκB. In porcine vascular smooth muscle cells, it has been demonstrated that down-regulation of O-GlcNAcylation mediated by OGA over-expression inhibits hyperglycemia-induced NFκB activation. In contrast, an increase in O-GlcNAcylation mediated by OGT over-expression increases NFκB activity (41). These effects were due to an increase in O-GlcNAcylation of RelA on T352 that decreases its affinity for IκB, leading to an increased nuclear translocation of RelA [Figure 2A (I)]. This could contribute to the sustained activation of NFκB that is associated with diabetes (41). Another study indicated that O-GlcNAcylation increases NFκB transcriptional activity by promoting its acetylation (42). Indeed, chromatin immunoprecipitation assays demonstrated that, upon induction with TNFα, OGT localizes to NFκB-regulated promoters. OGT siRNA experiments showed that OGT protein was required for NFκB-dependent transcription. The mechanism involved was the attachment of *O*-GlcNAc moiety to T305 on RelA that promoted NFκB transcription by potentiating p300-dependent acetylation on K310 [Figure 2A (II)] (42).

The O-GlcNAcylation of NFκB also appears to play an important role in the immunity and the production of pro-inflammatory cytokines by T lymphocytes. Golks et al. first showed that OGT was necessary for activation of T lymphocytes by the T-cell receptor (TCR), inducing O-GlcNAcylation of p65 and stimulation of NFκB-dependent transcription (47). More recently, it was reported that in these cells, the c-Rel subunit of NFκB was modified by O-GlcNAcylation on Ser 350 [Figure 2A (III)]. This modification increased c-Rel transcriptional activity and was necessary for c-Rel mediated expression of *IL2, IFNG*, and *CSF2* in response to TCR activation (48). Importantly c-Rel O-GlcNAcylation was not required for TNFα- or TCR-induced expression of other NFκB target genes, such as *NFKBIA* (which encodes IκBα) and *TNFAIP3* (which encodes A20), indicating a gene specific requirement of c-Rel O-GlcNAcylation (48). These results suggest that during chronic hyperglycemia, an increase in c-Rel O-GlcNAcylation could contribute to type-1 diabetes progression by enhancing the production of Th1 pro-inflammatory cytokines, leading to pancreatic β cells destruction (48, 49). Finally, O-GlcNAcylation of IKK [Figure 2A (IV)] has also been demonstrated, resulting in an increase in its kinase activity, leading to subsequent increase in phosphorylation, and degradation of IκB and stimulation of NFκB activity in cancer cells (50). Whether this mechanism is also operative in the context of hyperglycemia-induced inflammation remains to be evaluated.

### O-GlcNAcylation as a negative regulator of NFκB activity

Whereas O-GlcNAcylation is generally found associated with an increased in NFκB activity in diabetic conditions, in some situations, *O*-GlcNAc appears, however, to reduce its pro-inflammatory activity (51–53). Thus, in a rat model of trauma-hemorrhage followed by fluid resuscitation, increased O-GlcNAcylation induced by glucosamine or PUGNAc significantly improved cardiac function and peripheral organ perfusion, and decreased the circulating levels of pro-inflammatory cytokines TNFα and IL6 (51, 52). These authors observed that increased O-GlcNAcylation reduces IκB phosphorylation and NFκB signaling in cardiac tissue from trauma-hemorrhage treated rats. Moreover, O-GlcNAcylation-inducing treatments appear to have anti-inflammatory and vaso-protective effects during acute vascular injury (54, 55). Indeed, Xing et al. showed that in rat aortic smooth muscle cells, O-GlcNAcylation of p65 NFκB upon PUGNAc or glucosamine treatment was accompanied by a reduction in TNFα-induced phosphorylation on serine 536, resulting in increased association of NFκB with IκB, decreased NFκB activity and inhibition of the production of pro-inflammatory mediators (Figure 2B) (53).

It therefore appears that, depending on the cellular context and type of insult (chronic hyperglycemia versus acute vascular injury), O-GlcNAcylation may have different effects on the NFκB pathway, resulting in either pro- or anti-inflammatory outcomes.

## O-GlcNAcylation and Macrophage Activity

Monocytes and macrophages play central roles in acute and chronic inflammatory processes. As mentioned previously, insulin resistance, obesity, and diabetes are associated with recruitment of pro-inflammatory monocytes/macrophages in different organs, including adipose tissue, liver, pancreas, as well as blood vessels wall (56–62). Numerous studies have shown that macrophages/monocytes submitted to long-term exposure to high-glucose concentrations developed a pro-inflammatory phenotype. Indeed, in human monocytic cells THP1, high glucose (15 mmol/L) for 72 h increased gene expression of the pro-inflammatory factors monocyte chemotactic protein 1 (MCP1), IL1β, and TNFα. Of interest, in this study, the NFκB activation played an important role in the high glucose-induced MCP1 transcription (63). In THP1 cells, exposure to high glucose also increased the RNA and protein levels of TLR2 and TLR4, which play key roles in innate immune response and inflammation. TLR2 and TLR4 activate MyD88 dependant signaling and induce NFκB transactivation, leading to the production of pro-inflammatory cytokines. These up-regulations of TLR2 and TLR4 under high-glucose condition seemed at least in part mediated by protein kinase C (PKC) (64). In RAW 264.7, a murine macrophages cell line, high-glucose alone did not induce inflammatory mediator expression but increased inducible nitric oxide synthase (iNOS) expression and nitric oxide (NO) production in response to LPS. This effect appeared to be mediated by NFκB activation (65). High-glucose also increased IL1β secretion from LPS activated macrophages, a risk factor in diabetes that contributes to pancreatic β-cell damage (66). This effect appeared to involve activation of ERK1/2, JNK1/2, and PKCα and δ in macrophages cultured in high-glucose conditions (65).

*In vivo* hyperglycemia also affects the inflammatory profile of macrophages. An increased pro-inflammatory profile was observed in peritoneal macrophages from mice two weeks after diabetes induction with alloxan or streptozocin (67, 68). However, peritoneal macrophages from mice with 4 months streptozotocin-induced diabetes displayed complex modification of the pro-inflammatory profile, with increased NO production but decreased TNFα and IL6 in response to LPS stimulation (69). Another study showed impaired inflammatory response to multiple TLR ligands in alveolar macrophages from 2 weeks streptozotocin-induced diabetic mice (70). Therefore, *in vivo* hyperglycemia may have complex effects on macrophages functions, depending on their tissue of origin and on the duration of the diabetes.

High-glucose concentrations may affect macrophages functions through numerous mechanisms, including oxidative stress, activation of PKC, and/or MAP kinases, advanced glycation end products, as well as protein O-GlcNAcylation. Only a few studies evaluated the role of O-GlcNAcylation in macrophages functions, and contradictory results were obtained.

In the human monocyte THP1 cell line, high-glucose concentrations and PUGNAc increased the expression and the secretion of macrophage inflammatory protein MIP1α and β through OGT dependent epigenetic mechanisms (71).

On the other hand, glucosamine exerted neuroprotective effects via suppression of post-ischemic microglia inflammation in rat brain after ischemia/reperfusion injury (72). Accordingly, in cultured mouse BV2 microglial cells and RAW264.7 macrophages, Hwang et al. observed that glucosamine suppressed LPS-induced up regulation of pro-inflammatory molecules by inhibiting NFκB activation by LPS. Glucosamine, which bypass the rate limiting step of the HBP, is often used to increase O-GlcNAcylation in cells. Unexpectedly, in this study, glucosamine induced a decrease in NFκB O-GlcNAcylation. This counter-intuitive result was explained by an inhibitory effect of glucosamine on an LPS-induced interaction between OGT and NFκB (72). More recently, the same group obtained similar results with cRel in BV2 microglial cells, showing glucosamine inhibition of LPS-induced cRel-OGT interaction, associated with decreased O-GlcNAcylation of c-Rel and subsequent inhibition of its transcriptional activity (73). However, the mechanism by which glucosamine may interfere with the LPS pathway and affect OGT-NFκB interaction was not elucidated. For instance, the specific effect of increasing O-GlcNAcylation levels using PUGNAc or Thiamet-G was not evaluated in theses studies. Glucosamine, by increasing UDP-GlcNAc in the cell, may also affect complex glycosylations of proteins. Thus, it is possible that glucosamine effects were mediated by modification of N-linked glycosylation of receptors and/or secreted proteins, as suggested previously in a study using macrophage cell lines (74). Moreover, depending on the experimental setting, glucosamine may also induce ATP depletion (75) or promote oxidative stress (76). Therefore, glycosylation-independent effects might also play a role in the paradoxical effect of glucosamine on NFκB O-GlcNAcylation state. Further confusion was provided by an additional study by Hwang et al. (77), which showed that over-expression of OGT unexpectedly reduced the transcriptional activity of NFκB both in the absence and presence of glucosamine, resulting in inhibition of LPS-mediated expression of the NFκB target gene iNOS.

Innate immune signaling initiated by interaction of pathogen ligands with TLRs induces iNOS expression, and, subsequently, the production of NO, which not only plays a role as a bactericidal agent but also act as an intracellular mediator. Indeed, S-nitrosylation of cysteine thiols regulates protein activities in NO-generating cells. Complex interactions between NO signaling and O-GlcNAcylation pathway have been suggested. Thus, in RAW264.7 cells and in mice peritoneal macrophages, Ryu et al. observed that LPS treatment induces increased global S-Nitrosylation of proteins, concomitant with a paradoxical denitrosylation of *S*-nitrosylated OGT (78). Denitrosylation of OGT was associated with an increase in its catalytic activity, suggesting a potential mechanism for LPS-induced O-GlcNAcylation of p65 and subsequent production of pro-inflammatory cytokines (78). On the other hand, in N9 microglia cells, Zheng et al. observed that LPS induced a (modest) reduction in global O-GlcNAcylation of proteins, associated with a reduction in OGT protein level (79). Clearly, additional work will be needed in order to untangle the complex relationships between OGT and p65 and their potential regulation by LPS, glucosamine, and S-nitrosylation signaling pathways, and to firmly establish their relative role in pro- and anti-inflammatory functions in macrophages.

## Conclusion

Whereas the implication of hyperglycemia in metabolic syndrome-associated inflammation is now well established, the involvement of O-GlcNAcylation appears complex, with both pro- and anti-inflammatory effects associated with this modification, depending on the type and duration (acute versus chronic) of the insult (80). In agreement with a dual effect of *O*-GlcNAc on inflammation, O-GlcNAcylation of NFκB, through an array of different mechanisms, can have both positive and negative effects on its activity depending on pathophysiological models and cell types (31, 41, 42, 47, 48, 51, 52, 81).

Recent data suggested that O-GlcNAcylation in the immune system may participate in the pathogenesis of both type-1 and type-2 diabetes (48, 49). Interestingly, O-GlcNAcylation was discovered 30 years ago in immune cells (82), and dynamic changes in *O*-GlcNAc levels upon lymphocyte activation were detected as early as the beginning of the nineties (83). However, only a limited amount of studies have investigated the function and regulation of this modification in immune cells, and very few works concern macrophages biology. This is indeed an emerging field, with many deficiencies in the existing knowledge. Several important points should be addressed in the future. Thus, the role of OGT and O-GlcNAcylation on macrophage functions (phagocytosis, ROS production in the phagosome, cytokine expression and secretion, M1 versus M2 polarization, etc.) should be thoroughly investigated. Ideally, these studies should be performed using primary cultured macrophages rather than in cell lines. In addition, the consequences of *in vivo* chronic hyperglycemia on protein O-GlcNAcylation in macrophages should also be evaluated. In this context, the development of macrophages specific OGT or OGA knock-out mice should provide important clues on the role of this modification in hyperglycemia-induced inflammation. Therefore, a large continent in the *O*-GlcNAc world remains to be explored.

## Conflict of Interest Statement

The authors declare that the research was conducted in the absence of any commercial or financial relationships that could be construed as a potential conflict of interest.
